# Healthcare resource utilization in Hepatitis C-infected patients completing eight versus twelve weeks of treatment: A retrospective cohort study

**DOI:** 10.3389/fgstr.2022.988971

**Published:** 2022-09-15

**Authors:** Katia E. Valdez, Marjan Javanbakht, Kori Keith, Roxanne Archer, John Z. Deng, Steven E. Marx, Arina Kuznetsova, Douglas E. Dylla, Jeffrey D. Klausner

**Affiliations:** ^1^ Feinberg School of Medicine, Northwestern University, Chicago, IL, United States; ^2^ Department of Epidemiology, University of California Los Angeles Fielding School of Public Health, Los Angeles, CA, United States; ^3^ Department of Population and Public Health Sciences, University of Southern California Keck School of Medicine, Los Angeles, CA, United States; ^4^ David Geffen School of Medicine, University of California Los Angeles, Los Angeles, CA, United States; ^5^ Global Scientific Publications (GSP) Specialty Medicine-Hepatology, AbbVie, Inc., Mettawa, IL, United States

**Keywords:** Hepatitis C Virus (HCV), resource utilization, healthcare encounters, sustained virologic response (SVR12), treatment regimen

## Abstract

**Background:**

The objective of this study was to examine differences in healthcare utilization among patients receiving eight vs. 12-weeks of treatment for infection with the Hepatitis C Virus (HCV).

**Methods:**

We conducted a retrospective cohort study among 282 treatment-naïve, HCV-infected patients. Those eligible were uninfected with the Human Immunodeficiency Virus, non-cirrhotic, and treated between 2016 and 2019 as part of an extensive, urban, university-affiliated healthcare system. Electronic medical data were abstracted starting from HCV treatment initiation and up to one year post-initiation or achievement of post-treatment sustained virologic response, whichever occurred first. The primary outcome of interest was healthcare utilization, defined by the number and type of healthcare encounters. Differences in healthcare utilization between those receiving eight vs. 12-weeks of treatment were examined using Student’s t-test, Fisher’s exact test, Pearson’s chi-square test, and the Wilcoxon rank-sum test.

**Results:**

A total of 282 eligible patients were analyzed. At baseline, the average age was 59 years (standard deviation=12), and the majority were male (55%) and white/Caucasian (58%). There were no baseline demographic or clinical differences between those completing 8 (n=59) or 12 (n=223) weeks of treatment. While no overall difference in healthcare encounters was observed between those receiving the 8-weeks (median encounters 6; IQR 4-11) and 12-weeks of treatment (median encounters 8; IQR 5-12; *P* value=0.07), a notable difference was seen in the number of laboratory visits between the groups (median 1 vs. 2; P value=0.04).

**Conclusions:**

Our findings indicate modest reductions in healthcare utilization among those receiving shorter treatment regimens for HCV infection, specifically regarding laboratory testing. These findings suggest that shorter treatment regimens may improve treatment expansion in settings that are otherwise too resource-constrained to deliver HCV care successfully.

## Background

The implementation of direct-acting antivirals for the treatment of Hepatitis C Virus (HCV), beginning with their Food and Drug Administration approval in 2011 (1), was a major clinical breakthrough that resulted in a reduction in the burden of disease among those infected with HCV (2). These new treatments have significantly increased the number of patients successfully cured, and shorter regimens have been shown to reduce the overall cost of treatment while achieving comparable rates of sustained virologic response (SVR) to the 12-week course (2, 3). Current guidelines recommend an eight or 12-week pan-genotypic treatment for HCV treatment-naïve patients with and without cirrhosis (4, 5). Beginning with the approval of ledipasvir/sofosbuvir (LDV/SOF) in 2014, some HCV treatment-naïve patients became eligible for this shortened 8-week treatment duration (3, 6, 7). Approval of the pan-genotypic HCV regimen glecaprevir/pibrentasvir (G/P) in 2017 (8) expanded patient eligibility for these shortened 8-week treatments (2, 5–7).

Previous studies have measured the healthcare costs associated with length of treatment, including expenses related to HCV treatment in those dually infected with Human Immunodeficiency Virus (HIV) (9–11). Shorter treatment times may critically reduce the financial impact on vulnerable patients and allow savings to both payors and hospital systems. Despite these treatment innovations, data on the real-world implementation of the shorter 8-week treatment regimen assessing the clinical and economic benefits are limited. To our knowledge, no study has reviewed the differential impact of varying treatment durations on healthcare utilization, and there are few data on these differences analyzed by type of healthcare encounter. To address this gap, our study aimed to measure healthcare resource utilization, by encounter type, in a retrospective cohort study of HCV-infected, non-cirrhotic, HIV-uninfected, and treatment naïve patients completing either eight or 12-weeks of therapy.

## Methods

### Study design

**Figure 1:**
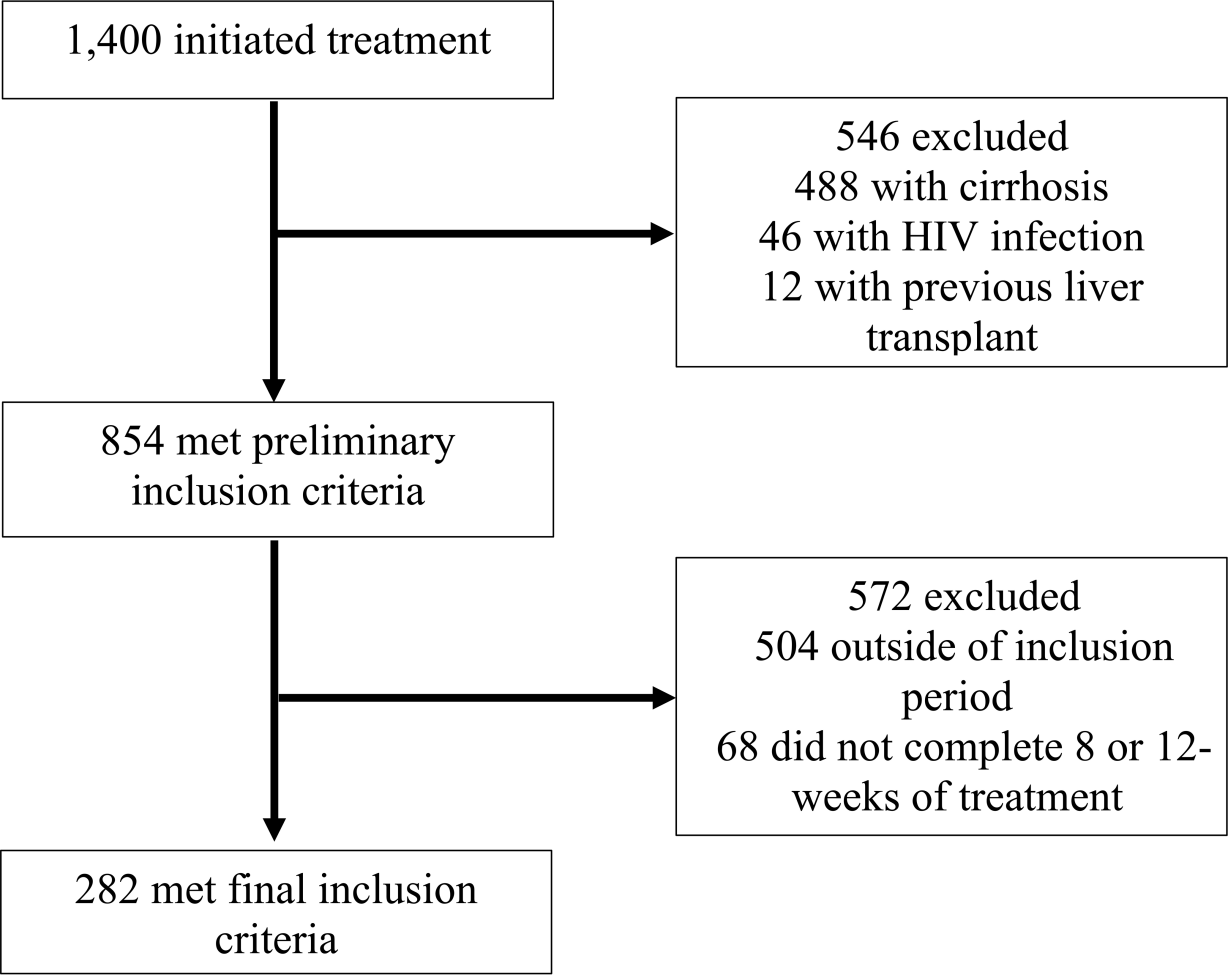
Flow diagram of study medical record inclusion between January 1, 2016, and January 1, 2019, University of California Health, Los Angeles.

This was a retrospective cohort study of patients receiving HCV care at an extensive, university-based, urban healthcare system in Los Angeles, CA. Eligible patients were 18 years of age or older, HCV-treatment naïve, initiated treatment at any clinic within the health system between January 1, 2016, and January 1, 2019, and completed either an eight or 12-week course of HCV treatment. Patients were excluded from the study if they were shown to have liver cirrhosis, co-infection with HIV, unsuccessful treatment of Hepatitis C, or had previously undergone a kidney or liver transplant. Liver cirrhosis was defined as a previously confirmed biopsy, a FibroScan score greater than or equal to 12kPa (12), a FIBROSpect score of greater than or equal to 42 (13), or a FIB-4 score of greater than 5.88 (14). Among the 1,400 patients who initiated HCV treatment in the potential study population, 282 met inclusion criteria and were included in this study. Details of the case-patient inclusion and exclusion are illustrated in **Figure 1**.

### Study outcomes and covariates

Data were abstracted from the electronic health records, and patients were followed starting with HCV treatment initiation. All healthcare actions and interactions occurring on and after the initiation date were recorded until sustained viral response at 12 weeks (SVR12) was assessed or up to one year after the initiation of treatment, whichever occurred first. Baseline demographic characteristics for each patient were captured from the most recent documented value before treatment initiation. Captured demographic characteristics included: age (in years) at the initiation of treatment, race and ethnicity, sex assigned at birth, and year of HCV treatment initiation. Baseline laboratory tests, values, and results included HCV genotype and sub-genotype (1, 1a, 1b, 2, 2a, 2b, 3, 4, 5, 6) (15), HCV RNA viral load, aspartate aminotransferase, alanine aminotransferase, total bilirubin, albumin, serum platelet count, and serum creatinine level. Other baseline laboratory testing data collected included a comprehensive metabolic panel, a complete blood count, and the prothrombin time test panel. Baseline viral Hepatitis tests collected included the Hepatitis B viral DNA, Hepatitis B surface antibody, and Hepatitis B core antibody. Baseline tests collected for the severity of liver disease included a liver biopsy, Fibrotest, FibroScan, FIBROSpect, magnetic elastography, and shear-wave elastography.

The number and type of clinical visits, phone calls logged, laboratory tests, procedures, provider notes, orders, and clinical support instances relating to a patient’s HCV treatment were abstracted from the electronic health record to determine healthcare utilization per patient throughout their HCV treatment. The electronic health record system has a category for visit type, which was used to determine the number of encounters relevant to HCV documented during the study period.

*Clinical Support* was defined as medication notifications, patient referrals, and treatment updates. *Laboratory Visits* were defined as a physical visit for medical test collection and procedures performed relating to HCV treatment and medication use. Multiple laboratory tests may have been performed during a single laboratory visit. *Notes & Orders* were defined as notes from a physician, nurse, or any other clinical staff regarding treatment, orders placed on behalf of treatment, the measurement of HCV viral load, or procedures for determining treatment efficacy. An *Office Visit* was defined as an in-person or telehealth visit to or with the physician’s office or hospital center for treatment prescription or medication follow-up. Lastly, *Communications* were defined as telephone calls to or from the patient regarding medication or messages sent between the patient and the healthcare system, utilizing the provided online patient portal.

### Analysis

Descriptive statistics of the mean, median, range, interquartile range, and frequency distribution were performed at baseline. Differences between the eight and 12-week treatment duration groups were evaluated using chi-square and Wilcoxon rank-sum tests for non-parametric samples. A Wilcoxon rank-sum test for non-parametric samples and the median and interquartile range were utilized in our final analysis. All analyses were performed using SAS 9.4 (Cary, NC).

The primary outcome of interest was healthcare resource utilization, which was measured as the total healthcare encounters per patient throughout their treatment. Encounters facilitating treatment for each patient, including physician, nurse, and pharmacist completion of clinical visits, phone calls, laboratory testing, and imaging studies, were measured. These encounters were abstracted under five categories: *Clinical Support, Laboratory Visits, Notes & Orders, Office Visits*, and *Communications*. The index date was the date of HCV treatment initiation, and the end of follow-up was defined as achievement of SVR12 or one year post-initiation of treatment, whichever occurred first. All eligible patients in both treatment cohorts were successfully cured of HCV infection. Differences in the total healthcare utilization and by clinical encounter type comparing eight vs. 12-week therapy were evaluated using parametric and non-parametric tests, as appropriate.

## Results

### Characteristics of the sample

The demographic characteristics of patients by treatment group are summarized in **Table 1**. Among the 282 patients meeting eligibility criteria, 59 (21%) completed 8-week treatments, and 223 (79%) completed 12-week treatments. Overall, the average age at treatment initiation was 59 years, more patients were male (55%), and white/Caucasians comprised the most prominent race/ethnicity group (58%). The most common genotype across both eight and 12-week groups was genotype 1, comprising 69.5% of the 8-week and 63.2% of the 12-week group. Genotypes 4 and 6 were grouped into the *Other* category to avoid empty data cells for chi-square analysis and made up 0% and 5.4% of the eight and 12-week groups. Within the 8-week group, there were no patients with genotypes 4 and 6; patients with genotypes 4 and 6 comprised 3.6% and 1.8% of the 12-week group, respectively. No one in the 8-week group had Hepatitis B DNA present at baseline, while 0.9% of the 12-week group had DNA present. The year of initiating HCV treatment was not statistically different between the eight and 12-week groups.

**Table 1:** Frequency of demographic characteristics of patients treated for Hepatitis C Virus infection between January 1, 2016, and January 1, 2019, University of California Health, Los Angeles.

Treatment Duration				
	Total*	8-Week	12-Week	
Patient Characteristics	N = 282	N = 59	N = 223	*P* Value†
***Age at Treatment Initiation in Years*,** *Mean (Standard Deviation)*	59.1 (12.0)	58.7 (12.8)	59.2 (11.8)	0.74
***Sex***				
Female	126 (44.7%)	23 (40.0%)	103 (46.2%)	0.32
Male	156 (55.3%)	36 (61.0%)	120 (53.8%)	---
***Race/Ethnicity***				
White (not Hispanic)	164 (58.2%)	42 (71.2%)	122 (54.7%)	0.12
Black (not Hispanic)	33 (11.7%)	6 (10.2%)	27 (12.1%)	---
Hispanic (any race)	40 (14.2%)	4 (6.8%)	36 (16.1%)	---
Other ‡	33 (11.7%)	5 (8.5%)	28 (12.6%)	---
***Viral Genotype***				
1	182 (64.5%)	41 (69.5%)	141 (63.2%)	0.28
1 unspecified	9 (3.2%)	3 (5.1%)	6 (2.7%)	---
1a	124 (44.0%)	27 (45.8%)	97 (43.5%)	---
1b	49 (17.4%)	11 (18.6%)	38 (17.0%)	--
2	43 (15.2%)	7 (11.9%)	36 (16.1%)	---
2 unspecified	9 (3.2%)	1 (1.7%)	8 (3.6%)	---
2a	8 (2.8%)	1 (1.7%)	7 (3.1%)	---
2b	26 (9.2%)	5 (8.5%)	21 (9.4%)	---
3	25 (8.9%)	4 (6.8%)	21 (9.4%)	---
3 unspecified	22 (7.8%)	2 (3.4%)	20 (9.0%)	---
3a	3 (1.1%)	2 (3.4%)	1 (0.4%)	---
Other§	12 (4.3%)	0 (0%)	12 (5.4%)	---
4a	8 (2.8%)	0 (0%)	8 (3.6%)	---
6a	4 (1.4%)	0 (0%)	4 (1.8%)	---
***Viral Hepatitis B Status***				
Hepatitis B DNA Present	2 (0.7%)	0 (0%)	2 (0.9%)	0.47
Hepatitis B Surface Antibody (Positive)	54 (19.1%)	11 (18.6%)	43 (19.3%)	0.91
Hepatitis B Core Antibody (Positive)	21 (7.4%)	5 (8.5%)	16 (7.2%)	0.74
***Year of Treatment Initiation***				
2016	137 (48.6%)	24 (40.7%)	113 (50.7%)	0.37
2017	82 (29.1%)	19 (32.2%)	63 (28.3%)	---
2018	63 (22.3%)	16 (27.1%)	47 (21.1%)	---

*Sums may be less than the total due to missing values.

†P values represent the comparison between 8 and 12-week treatment duration groups. ‡Other category includes Asian, American Indian, Native Hawaiian or Other Pacific Islander, and Native Alaskan.

§Other category includes genotypes 4 and 6.

### Characteristics of the baseline clinical tests

The frequency of clinical tests and panels performed at baseline by treatment group are summarized in **Table 2**; significant differences were seen between groups in the frequency of liver biopsies and prothrombin time test panels performed at baseline (i.e., before treatment initiation). Significantly more patients in the 12-week group (29.1%) received a liver biopsy to test for the severity of liver disease as compared to the 8-week group (11.9%) (*P*=0.007). However, no significant differences were seen in the distribution of the year that the biopsies were performed between the two groups (*P*=0.97). Fewer than 50% of overall patients received a Fibrotest, Fibroscan, shear-wave, or magnetic elastography at baseline. No differences were seen between the frequency of Hepatitis B tests performed at baseline. More patients in the 12-week group received a baseline Prothrombin Time test panel (89.2%) as compared to those in the 8-week group (79.7%) (*P*=0.05).

**Table 2:** Frequency of clinical tests and panels performed at baseline among patients treated for Hepatitis C Virus infection between January 1, 2016, and January 1, 2019, University of California Health, Los Angeles.

		Treatment Duration		
	Total*	8-Week	12-Week	
Tests Performed	N = 282	N = 59	N = 223	*P* Value†
***Tests for Severity of Liver Disease***				
Liver Biopsy‡	72	7 (11.9%)	65 (29.1%)	<0.01
Fibrotest	19	6 (10.2%)	13 (5.8%)	0.24
Fibrospect	132	30 (50.8%)	102 (45.7%)	0.48
Fibroscan	7	2 (3.4%)	5 (2.2%)	0.61
Elastography	23	4 (6.8%)	19 (8.5%)	0.66
***Viral Hepatitis Tests***				
Hepatitis B DNA	31	6 (10.2%)	25 (11.2%)	0.82
Hepatitis B Surface Antibody	144	36 (61.0%)	108 (48.4%)	0.09
Hepatitis B Core Antibody	85	22 (37.3%)	63 (28.3%)	0.18
***Blood Panels***				
Comprehensive Metabolic Panel§	260	55 (93.2%)	205 (91.9%)	0.74
Complete Blood Count¶	272	56 (94.9%)	216 (96.9%)	0.47
Prothrombin Time Test#	246	47 (79.7%)	199 (89.2%)	0.05

*Sums may be less than the total due to missing values.

† P values represent the comparison between 8 and 12-week treatment duration groups.

‡There were no significant differences in the distribution of the year the biopsy performed between those completing 8 and 12 weeks of HCV treatment (P = 0.97).

§Comprehensive Metabolic Panel includes: Glucose, Calcium, Sodium, Potassium, Carbon Dioxide, Chloride, Albumin, Total Protein, Alkaline Phosphatase, Alanine Aminotransferase, Aspartate Aminotransferase, Bilirubin, Blood Urea Nitrogen, Creatinine.

¶Complete Blood Count includes: Red Blood Cell Count, Hemoglobin, Hematocrit, White Blood Cell Count, Platelet Count.

#Prothrombin Time Test includes: Prothrombin Time, International Normalized Ratio.

### Characteristics of healthcare encounters

**Figure 2:**
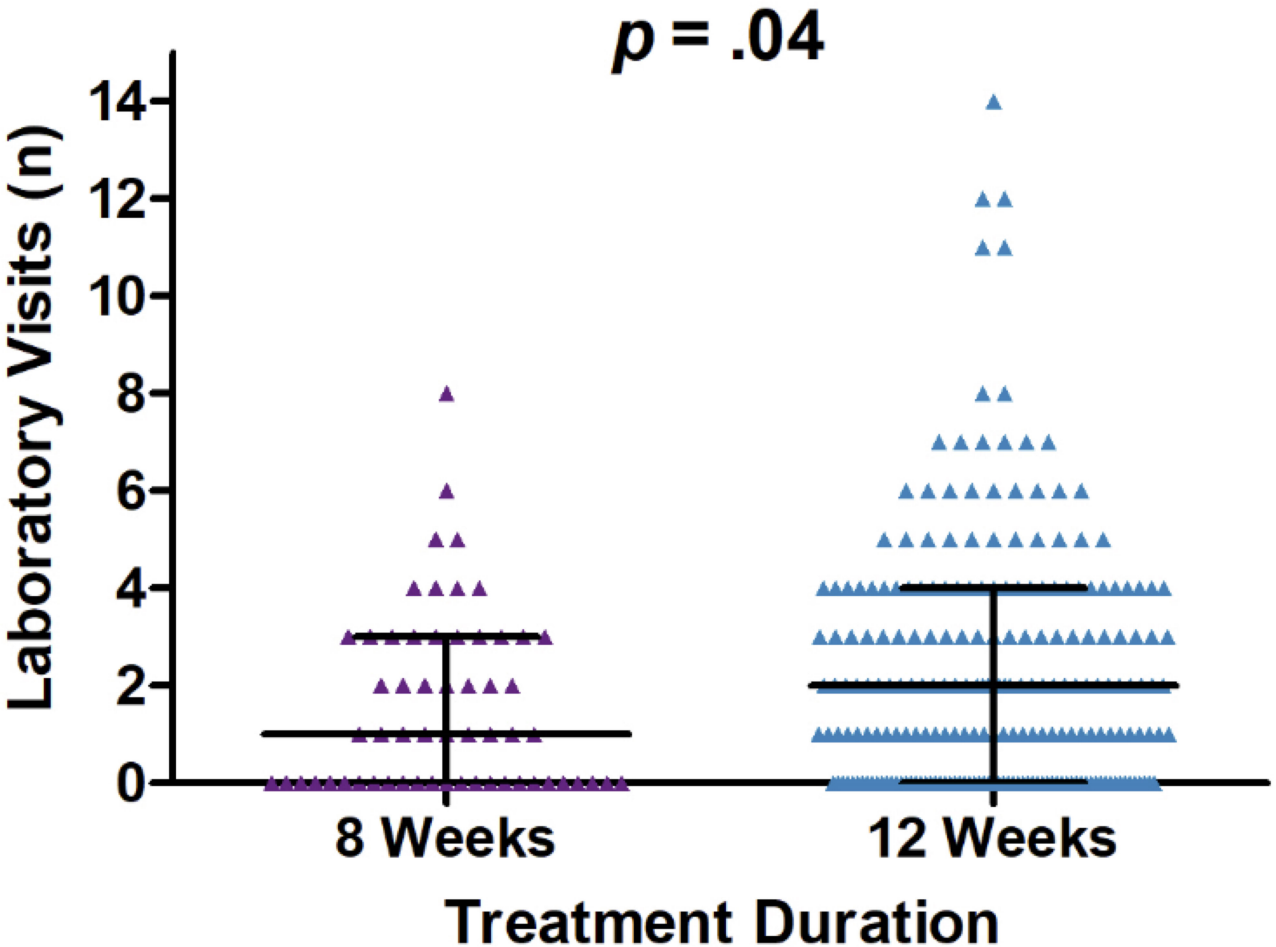
Dot plot of laboratory visit encounters occurring on and after the initiation date and until sustained viral response at 12 weeks (SVR12) or up to one year after initiation of treatment, whichever occurred first. Encounters occurred between January 1, 2016, and January 1, 2019, University of California Health, Los Angeles.

Comparisons of the number of healthcare encounters by encounter type are summarized in **Table 3**. Overall, the median number of healthcare encounters throughout treatment was 8 [interquartile range (IQR) 5-12] for those receiving a 12-week treatment regimen as compared to 6 (IQR 4-11) for those on an 8-week treatment regimen (*P*=0.07). Fewer laboratory visits were utilized in the 8-week group, with an average of 1.6 visits (median=1) as compared to 2.3 visits (median=2) in the 12- week group (*P*=0.04; for comparison of medians) (**Figure 2**). No differences were noted in the number of clinical support encounters, notes and orders, office visits, and communications encounters across groups.

**Table 3:** Analytic description of the number of encounters per individual by type, among patients treated for Hepatitis C Virus infection between January 1, 2016, and January 1, 2019, University of California Health, Los Angeles*.

Treatment Duration							
Encounter Type†	8-Week (N=59)			12-Week (N=223)			
Mean (SD)	Median (IQR)	Range	Mean (SD)	Median (IQR)	Range	*P* Value‡
Any Encounter	9.0 (8.6)	6 (4-11)	1-55	9.9 (7.8)	8 (5-12)	1-55	0.07
Clinical Support	1.0 (1.6)	0 (0-1)	0-8	0.7 (1.0)	0 (0-1)	0-6	0.24
Laboratory Visits§	1.6 (1.8)	1 (0-3)	0-8	2.3 (2.5)	2 (0-4)	0-14	0.04
Notes & Orders	1.6 (3.5)	0 (0-1)	0-21	1.8 (2.5)	1 (0-2)	0-14	0.06
Office Visits	2.4 (1.9)	2 (1-3)	1-10	2.4 (2.1)	2 (1-3)	1-13	0.80
Communications	2.4 (4.1)	1 (0-3)	0-20	2.7 (4.9)	1 (0-3)	0-33	0.46

*Total represents cumulative number of encounters across all patients.

†Emergency Department Visits, Patient Visits, and Hospital Admission encounters were excluded.

‡P values represent the comparison between 8 and 12 week treatment duration groups, Wilcoxon rank-sum test.

§Encounters exclude tests performed at baseline.

Clinical Support: Medication notifications, patient referrals, treatment updates.

Laboratory Visit: Total number of physical visits for medical test collection and procedures related to medication use. Multiple tests may have been performed during a single laboratory visit.

Notes & Orders: Notes from a physician, nurse, or any other clinical staff regarding treatment. Orders placed on behalf of treatment, measurement of viral load, or procedures for determining the efficacy of treatment.

Office Visit: In-person or telehealth visit to the office or with the provider for treatment prescription or medication follow up.

Communications: Telephone calls to or from the patient regarding medication, or messages sent between the patient and the healthcare system which utilized the provided online patient portal.

## Discussion

Our findings indicate a moderate reduction in healthcare utilization among patients receiving a shorter treatment regimen for HCV infection. While the number of in-person clinical visits and other ancillary support visits were similar for both treatment duration groups, the 8-week treatment regimen resulted in significantly less laboratory testing following HCV treatment initiation.

Additionally, we found that patients receiving an 8-week treatment regimen had decreased testing before HCV treatment initiation, including testing for the severity of disease and liver cirrhosis, compared to the 12-week treatment group. Notably, patients undergoing the 12-week regimen had liver biopsies more frequently than those on the 8-week regimen. While this may suggest a differential clinical presentation between these two groups, our data revealed that both groups were otherwise clinically comparable treatment-naïve, non-cirrhotic HCV patients (16). It is possible, however, that other non-clinical factors – such as a patient’s degree of insurance coverage or their ability to access hospital resources – affected a provider’s prescription of one treatment duration or another. These possible factors may have similarly influenced a patient’s access to liver biopsy resources. It is also worth noting that this study outcome was based on the number of encounters and not on individual tests. Our data imply that increased healthcare utilization for 12-week treatments includes additional care before and during treatment. In addition, a previous study suggests that less than half of patients eligible for 8-week treatment received it – this may further contribute to increased resource utilization (17). This disparity may be partly driven by a combination of provider preference or practice and health insurance requirements favoring a 12-week regimen. System-level changes such as expanded insurance authorization for 8-week regimens may be crucial in developing uptake and reducing the utilization of healthcare resources (18–20).

Though similar across treatment groups, office visits and communications were the most utilized encounters. In addition, we found that encounters entailing notes and orders related to patient management were more frequent among those in the longer treatment group. This supports our findings on laboratory testing data, suggesting that longer treatment durations may require more follow-up. As a result, those in the longer duration groups require more provider clinical notes regarding treatment status or orders for additional lab work, tests, or procedures to monitor patient clinical status. Hospital admission and emergency room visits were *a priori* excluded in our analysis due to the ambiguity surrounding the connection between HCV treatment and those encounter types.

With the expansion of the Centers for Disease Control and Prevention screening recommendations in 2020 to include at least one HCV screening test for all adults, the United States can expect to see an increase in the identification of cases (21). The application of shorter treatment durations can save substantial time and resources as the number of treated cases increases. One ongoing study monitors the efficacy of minimal monitoring in the administration of HCV treatment. These promising results show comparable SVR-12 outcomes to current, more involved treatment practices while minimizing the number of resources required for treatment monitoring (22). Conclusions such as these go hand in hand with our results, supporting the elimination of HCV by implementing both patient and provider interventions and system-level changes that will promote more efficient HCV treatment plans, reduce healthcare utilization, and ultimately improve capacity to increase HCV treatment.

The findings of this study should be interpreted considering some limitations. Our results are based on data collected from electronic health records. As a result, any encounter information such as case management interactions between staff that were not included in the electronic health record would not have been captured as part of this analysis. Our data likely underestimate the actual frequency of healthcare utilization; however, we anticipate that this potential bias is non-differential by treatment duration. Even though our study used a 3-year time frame to include eligible patients, the approval of 8-week G/P by the U.S. Food and Drug Administration occurred at the start of our study period, likely contributing to fewer patients in the 8-week treatment groups. While time trends by year of treatment initiation did not reveal a differential between the two groups (data not shown), the smaller sample size limited statistical power. This precluded us from conducting analyses beyond the descriptive statistics provided as part of this manuscript. Finally, our data are based on patients receiving care as part of an extensive, well-resourced healthcare system and may not be generalizable to other settings. Nonetheless, this study provides novel information about resource utilization and the potential for expanded treatment capacity resulting from shorter duration treatments for HCV infection.

## Conclusions

While other studies have examined the outcome of healthcare resource utilization in terms of financial costs to HCV-infected patients (23) and the health system (24–26), our study is one of the first to explore differences in healthcare utilization by treatment duration. Our findings suggest that the use of shorter regimens among non-cirrhotic HCV patients, when clinically indicated, may decrease healthcare resource utilization. These reductions would chiefly impact those with inadequate engagement with healthcare or with challenges related to transportation and access to laboratory services.

## Data availability statement

The datasets presented in this article are not readily available as they were utilized under license for the current study. Requests to access the datasets should be directed to Katia Valdez, at katia.valdez@northwestern.edu.

## Author contributions

KV, KK and JD abstracted the data for analysis. KV analyzed the data, wrote the main manuscript text, and constructed **Tables 1**–**3**. KV and RA constructed **Figure 1**, and SM, AK, and DD constructed **Figure 2**. MJ and JK created the experimental design and significantly contributed to the analysis of data, writing of the manuscript, and creation of **Figure 1** and **Tables 1**–**3** throughout the study. All authors contributed to the study design, interpretation of data, and review of the final manuscript. All authors have read and approved the manuscript.

## Acknowledgments

We would like to thank Arash Shamsian and the UCLA ISS Care Connect team for their continued support throughout this project.

## Conflict of interest

SM, AK, and DD are employees of AbbVie and may hold stock/share options.

The authors declare that this study was funded by a contract provided by AbbVie (Project ID: H18.VirologyHCV.07-SR1808). AbbVie sponsored the study, contributed to its design, and participated in collecting, analyzing, interpreting the data, writing, reviewing, and approving the manuscript.

## Publisher’s note

All claims expressed in this article are solely those of the authors and do not necessarily represent those of their affiliated organizations, or those of the publisher, the editors and the reviewers. Any product that may be evaluated in this article, or claim that may be made by its manufacturer, is not guaranteed or endorsed by the publisher.

## Abbreviations

HCV, Hepatitis C Virus; HIV, Human Immunodeficiency Virus; EHR, Electronic Health Record; SVR12, Sustained Virologic Response at 12-Weeks Post Treatment; MINMON, Minimal Monitoring.
